# Use of auditory learning to manage listening problems in children

**DOI:** 10.1098/rstb.2008.0187

**Published:** 2008-11-04

**Authors:** David R. Moore, Lorna F. Halliday, Sygal Amitay

**Affiliations:** MRC Institute of Hearing ResearchUniversity Park, Nottingham NG7 2RD, UK

**Keywords:** language problems, communication problems, attention, perceptual learning, individual differences, cognitive skills

## Abstract

This paper reviews recent studies that have used adaptive auditory training to address communication problems experienced by some children in their everyday life. It considers the auditory contribution to developmental listening and language problems and the underlying principles of auditory learning that may drive further refinement of auditory learning applications. Following strong claims that language and listening skills in children could be improved by auditory learning, researchers have debated what aspect of training contributed to the improvement and even whether the claimed improvements reflect primarily a retest effect on the skill measures. Key to understanding this research have been more circumscribed studies of the transfer of learning and the use of multiple control groups to examine auditory and non-auditory contributions to the learning. Significant auditory learning can occur during relatively brief periods of training. As children mature, their ability to train improves, but the relation between the duration of training, amount of learning and benefit remains unclear. Individual differences in initial performance and amount of subsequent learning advocate tailoring training to individual learners. The mechanisms of learning remain obscure, especially in children, but it appears that the development of cognitive skills is of at least equal importance to the refinement of sensory processing. Promotion of retention and transfer of learning are major goals for further research.

## 1. Introduction

We define auditory learning as any measurable improvement in performance of a listening task that is produced by a period of stimulation. This stimulation need not be auditory or involve deliberate, specific training. For instance, we have demonstrated (Amitay *et al*. 2006*b*, 2008) that the performance of auditory tasks may be improved by training with non-auditory stimuli, and users of hearing instruments improve their ability to use those instruments without specific training (e.g. Fryauf-Bertschy *et al*. 1997). Performance improvement is usually the greatest on the trained task, but can also transfer to other, untrained task or stimulus conditions. The pattern of transfer between conditions may be used to infer information about the auditory system, such as the neural mechanisms by which changes are likely to occur (e.g. Wright & Fitzgerald 2001; Demany & Semal 2002; Amitay *et al*. 2006*b*). However, we examine here the proposition that the transfer of auditory learning can be used to improve listening and language skills of immediate practical usefulness. As most of the relevant research to date has been performed on children, specific maturational aspects of those skills, and the training used to improve them, are considered in detail. In the longer term, we expect further development of auditory learning applications to be based on laboratory studies in both adults and children, so we finish with a review of work, mainly from our own group, designed to reveal some fundamental rules of learning. The paper is written from a translational perspective and is not meant to be a theoretical or comprehensive review. References to other review material are provided where appropriate.

## 2. Applications of auditory learning in children

While there have been many and varied attempts to train hearing for over 100 years (see Kricos & McCarthy 2007), systematic scientific studies of auditory learning in children have been pursued only recently. The majority of studies have examined 5–12 year old children with language-based learning impairments (LLI, see §3). They have been motivated by the hypotheses that deficits in auditory processing might cause those impairments (Tallal 2004), and that adaptive auditory training would lead to improved auditory performance and, hence, remediation of LLI. An initial study in children with LLI by Merzenich *et al*. (1996), leading to the development of Fast ForWord (Scientific Learning Corporation 1998, http://www.scilearn.com/products/index.php), trained tone and phoneme discrimination for 6–9 hours in the context of computer games. By adaptively varying the timing of the stimulus presentation, the training improved the children's performance on tests of auditory temporal processing. In a second study (Tallal *et al*. 1996), a group of children with LLI were trained for four weeks (88–116 hours) using a mix of the same temporal discrimination computer games and other exercises involving listening to temporally modified speech. A control group performed similar tasks without adaptive training or temporal modification of the stimuli. Learning on the training tasks and transfer (‘generalization’) to speech perception and language comprehension abilities occurred in both groups, but the group trained adaptively and with modified speech improved more than the controls. Fast ForWord has since been developed into a number of versions for different ages and ability levels and is being used by typically developing as well as by language-impaired children.

The rationale and efficacy of Fast ForWord have been questioned by researchers for a number of reasons. First, it trains children to discriminate a range of different stimuli, from rapid tone sweeps and simple speech sounds to complex linguistic tokens including syllables, words and sentences. Additionally, it provides audio material incorporating modified speech to which the child listens without adaptive training. It is thus impossible to tell which aspects of the reported improvements in language abilities are due to which stimuli (Gillam 1999). Second, although current incarnations of Fast ForWord are less intensive than the initial prototype (Merzenich *et al*. 1996; Tallal *et al*. 1996), children are still required to do 25–50 hours of training (http://www.scilearn.com/products/elementary-products/fast-forword-language/index.php). This is a lot for children, and no evidence has been reported for the time course, or its relation to transfer, of this learning. If at least some transferable learning occurs rapidly (e.g. Amitay *et al*. 2006*b*), it is unclear why children are required to spend so much time doing Fast ForWord or, conversely, whether shorter periods of training could be equally effective. Third, the selection of training exercises was predicated on the idea that children with LLI have a specific auditory temporal processing impairment. Further studies (e.g. Amitay *et al*. 2002; Ramus *et al*. 2003; Halliday & Bishop 2006*a*) have indicated that auditory impairments accompanying LLI are not limited to temporal processing and that, consequently, the training may not address other processing deficiencies. Finally, two recent, large-scale, randomized-control trials (Cohen *et al*. 2005; Gillam *et al*. 2008) have failed to show a significant remedial benefit for Fast ForWord over other computer-assisted or therapist-centred language interventions.

The concerns raised about Fast ForWord reflect the central issues to be presented and discussed in this paper. It is widely accepted that auditory training can improve performance on the trained task (e.g. Amitay *et al*. 2005) and that, at least in adults, listeners who perform most poorly at the outset of training, will generally improve more than those who initially perform well. However, the most important translational questions are whether, and to what extent, the training transfers to improved everyday skills such as speech perception and language. A recent study by McArthur *et al*. (2008) has addressed these questions by examining the effect of four separate types of auditory training on auditory processing and language abilities in children with LLI. The children learned the task on which they were trained, but the learning did not transfer to language tasks, relative to an untrained group of typically developing children. While these results were interpreted by McArthur *et al*. (2008) as suggesting that only the complex linguistic component of Fast ForWord training transfers to improved language, they may also relate to substantial differences between the auditory training used by McArthur and the tone and simple speech tasks of Fast ForWord. However, a more general point is that, in this and several other studies, all experimental groups improved on some of the test (‘outcome’) measures. Those measures were not designed to be used repeatedly over the typically short periods spanning the duration of training. Their repeated use over such periods may lead to memorization of the training materials or, more intriguingly, to training effects in themselves.

This raises the second critical issue—the appropriate control for training studies. As above, Tallal *et al*. (1996) compared performance between an ‘experimental’ group, trained on adaptive temporal tasks and a ‘placebo’ intervention control group, trained on similar, non-adaptive tasks that did not depend on temporal processing. They showed that both groups improved on the test measures, but the experimental group improved more. In this design, it is likely that the ‘placebo’ group actively benefited from (i) performing (training on) the tests used to measure the effect of the training, (ii) the alternate tasks practiced during the training period, and (iii) increased interaction with the training assistants or use of computer games. Green & Bavelier (2007) have, for example, shown in adults that training on arcade-style computer games that are not designed to enhance visual skills (e.g. spatial resolution) can nevertheless do so. A third group that did not perform any training, a ‘waiting-room’ control, could have distinguished between these options, but even that group may have trained on or otherwise benefited from repeated exposure to the test materials.

**Figure 1:**
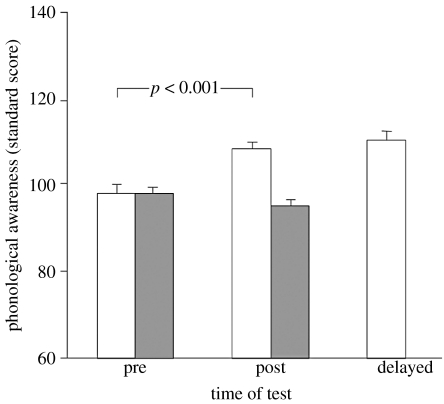
Improved language skills following phoneme discrimination training. Performance of trained (open square) and untrained (filled square) children before (pre), immediately after (post) and five to six weeks after (delayed) a four-week period during which the trained group performed phoneme discrimination exercises three times/week. Scores on the outcome measure (the four receptive sub-tests of the Phonological Assessment Battery; Frederickson *et al*. 1997) are referenced to the normalized (age-appropriate standard score) British values. Analysis of variance showed a highly significant training effect with no subsequent improvement (or decline) at the delayed test. The untrained group, who engaged in normal classroom activities while the other group trained, did not differ significantly between the pre and post tests. Data bars show group means. Error bars in all figures are standard errors. Adapted and modified with permission from Moore *et al*. (2005).

A study that used such an untrained, waiting-room control also addressed some of the other concerns about Fast ForWord and suggested the efficacy of auditory training for typically developing children (Moore *et al*. 2005). In this study (figure 1), 8- to 10-year-old typically developing children received just 6 hours of training on a phoneme discrimination task (Phonomena, MindWeavers plc, http://www.mindweavers.com/index.php) over four weeks. Compared with the untrained control group, children in the trained group showed a significant improvement in their phonological processing skills following training, and this improvement was maintained for at least five weeks following the cessation of training. In this instance, no improvement was seen in the control group as a result of retesting with the outcome measure, a standardized test of phonological awareness (Phonological Assessment Battery; Frederickson *et al*. 1997). These findings were taken by the authors to suggest that auditory (specifically, phoneme) discrimination training may be used to enhance the language abilities of typically developing children. However, unlike the Tallal *et al*. (1996) study, this study did not have a placebo control group. It is thus unclear whether the results were due to the auditory training itself or to other effects, as above. Another unanswered question in the Moore *et al*. (2005) study is whether the training required the specific type (phonemes) or modality (auditory) of stimuli used in the trained group. We are currently conducting further research to dissect these components of auditory training in children.

To our knowledge, no studies of auditory learning in children have used both waiting room and placebo trained control groups. This issue is taken up again in §4, but more applied research is needed to cast further light on the role played by different components of training, the effectiveness of different training stimuli and the time required to train.

## 3. Hearing and listening problems in children

To examine how auditory learning may remediate developmental hearing and listening problems in children, we consider in this section what is known about the nature of those problems.

### (a) Hearing and listening

Children hear well from an early age. The cochlea and brainstem are structurally and functionally well developed by six months and 2 years of age, respectively (Moore 2002), and most aspects of the perception of simple auditory stimuli are complete within the first few years (Werner & Gray 1998). However, whether gauged by cortical evoked (Ponton *et al*. 2000) or behavioural (Hartley *et al*. 2000) responses, we find that children's auditory function typically remains immature beyond 10 years of age. The major research issue in trying to understand this late development has been whether it reflects underlying sensory capacities, or a range of poorly segregated functions that have been variously attributed to ‘processing efficiency’ (Hartley & Moore 2002; Hill *et al*. 2004), ‘internal noise’ (Buss *et al*. 2006) and ‘attentiveness’ (Werner 1992; Moore *et al*. 2008*a*). In auditory science, a convention has emerged (e.g. Kiessling *et al*. 2003) of referring to the presumed lower level and passive perception of simple stimuli as ‘hearing’, whereas the addition of higher level, active processing converts the task into one of ‘listening’. While such a dichotomy is undoubtedly a simplification and lacks a cast iron evidence base, it does provide a convenient platform on which to consider a widespread problem of impaired ability in some children. As we (Halliday & Moore in press) and others (Bishop 2007) have detailed elsewhere, a large proportion (approx. 30–50%) of children diagnosed with a wide range of LLI (e.g. dyslexia) perform poorly on psychoacoustic tests of listening involving temporal and spectral resolution. The two issues to be addressed in the remainder of this section are, first, the nature of these listening problems—whether they are sensory or non-sensory in origin and, second, their relation to other hearing and language problems.

### (b) Auditory processing disorder

Auditory processing disorder (APD; also previously known as central, (C)APD and obscure auditory disorder) has been used as a clinical diagnosis for more than 30 years. Until quite recently, however, there has been little agreement about its definition, with a consequent diversity in the number of tests and treatments (Hind 2006; Moore 2006). Convergent operational definitions have now been provided by the US National Institutes of Health (see www.nidcd.nih.gov/health/voice/auditory.asp), the American Speech, Language and Hearing Association (see www.asha.org) and the British Society of Audiology (BSA; see www.thebsa.org.uk/apd). The new convergence centres on the hypothesis that APD is associated with poor performance on a range of basic listening skills, such as temporal and spectral resolution and discrimination, in the absence of audiometric insensitivity. Although APD has been described in adults, and particularly in adults with observable brain lesions (Bamiou *et al*. 2006), its prevalence is considered to be much higher in children. APD is thought by many auditory scientists and clinicians to underpin critical everyday listening difficulties, notably speech-in-noise perception and speech understanding in general (Chermak & Musiek 1997). Some think that these problems lead (causally) to LLI, but this link is currently controversial (e.g. Rosen 2003). Nevertheless, it is clear that a clinical demand exists for scientific insight into APD and children with listening problems are not currently receiving clear, scientifically based testing or management.

**Figure 2:**
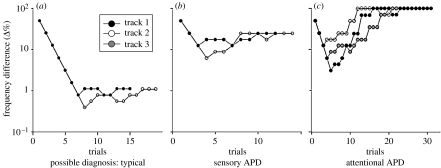
Types of listening performance in children. Each panel shows the results of successive, 3-down, 1-up staircase, adaptive tracks of trials in three individual children. The ordinate shows the frequency difference between the standard and target stimuli. (*a*) Good (typical) performers produced consistent responses at low threshold levels (black circle, track 1; white circle, track 2; grey circle, track 3). (*b*) Genuine poor performers were consistent, but had elevated thresholds. This behaviour was suggestive of a ‘sensory’ form of APD. (*c*) Non-compliant responders generally performed well in the first few trials of each track, but performance then declined, either to ceiling level (as here) or to a level close to, or above the starting level of the track. Performance often recovered towards the end of the track. This behaviour was suggestive of an ‘attentive’ form of APD. The examples shown are from (*a*) a 10-year-old, (*b*) a 9-year-old and (*c*) an 8-year-old child. Further details in the text. Adapted with permission from Moore *et al*. (2008*a*).

We have recently proposed two types of listening problem in 6- to 11-year-old children performing a pure-tone frequency discrimination involving adaptive staircase testing (Moore *et al*. 2008*a*). This classification was based on a quantitative analysis of, on the one hand, response threshold and, on the other, response variability (figure 2). Typical performance (figure 2*a*) was characterized by a ‘lead-in’ sequence in which easy-to-difficult discriminations produced a succession of correct responses. This resulted in a rapid approach to a consistent, threshold performance level that equalled or was close to that achieved by adults. A second test track typically had the same characteristics, indicating consistent, acute discrimination relative to others of the same age. ‘Genuine poor performers’ (figure 2*b*) had much less sensitive thresholds, but equally consistent responding both within and across tracks. This pattern was seen relatively rarely, particularly in younger children. A third pattern, which by contrast was seen in a larger number of especially younger children, was characterized both by poor thresholds and highly variable performance. These children often performed quite accurately and consistently during the lead-in trials, suggesting that they could both do the task and discriminate the stimuli. However, when they began to make mistakes during difficult discriminations, their performance declined, and they subsequently made mistakes for discriminations they had formerly achieved with ease. In a few extreme cases (figure 2*c*), they performed at ceiling but, more typically, their performance varied cyclically, with large excursions during the course of a test track. Their performance also often varied dramatically between tracks. We hypothesize that this behaviour, which we call ‘non-compliant’, is due to fluctuations of attention. However, nearly every paper that has been written on APD has emphasized the heterogeneous and/or multifaceted nature of the disorder, and it seems likely that other cognitive factors (e.g. working memory) contribute to non-compliant responding.

To test the relation between listening and cognitive performance of children further, we are currently conducting a large, multicentre study (Moore *et al*. 2008*b*) in which response threshold and variability on several tests of listening (frequency selectivity, temporal resolution and frequency discrimination) are quantified and correlated with measures of language, attention, memory, non-verbal IQ, reading and communication.

### (c) Relation to other hearing, listening and learning problems

In this section, we consider the extent to which APD overlaps with sensorineural hearing loss (SNHL) and LLI. SNHL is a permanent hearing loss caused by a defect in the cochlea or in the neural pathways from the cochlea to the brain. The extent of the loss varies considerably, from mild (defined here as a better ear pure tone threshold of 20–40 dB HL across 250–4000 Hz) to profound (more than 95 dB HL) (BSA 2004). Typically, children diagnosed with SNHL are provided with a hearing aid and/or cochlear implant, depending on the nature and the severity of the loss. However, the extent to which these hearing instruments serve to aid the hearing and/or listening problems of children with SNHL varies considerably from child to child.

Children with APD do not have SNHL, by definition. People with SNHL, on the other hand, have problems with listening, in addition to reduced sensitivity, that appear to be related to their peripheral pathology and that overlap substantially with the range of problems defined as symptomatic of APD. These include impaired frequency selectivity and discrimination, temporal resolution and integration and—especially for individuals with asymmetric losses—poorer spatial and binaural hearing (e.g. Halliday & Bishop 2005, 2006*b*; Moore 2007). However, there is evidence to suggest that some children with SNHL may also have problems in listening which are over and above those that would be predicted from their hearing loss. Research into children with cochlear implants has shown that there is considerable heterogeneity in the outcomes of this group, assessed both behaviourally and electrophysiologically (e.g. Sharma *et al*. 2002; Geers 2004; Hawker *et al*. 2008). This has led some researchers to argue that children might vary in the extent to which they can make use of the auditory information coming in through their device (Hawker *et al*. 2008; Pisoni *et al*. 2008). There is also some evidence that the presence of even a mild to moderate SNHL in childhood may require additional mental resources and effort during listening (for review, see Jerger 2007). These findings suggest that, in some cases, the presence of SNHL during childhood can lead to, or at least be associated with, listening as well as hearing difficulties.

LLI is an umbrella term that is commonly used to describe a heterogeneous group of children, including those with specific language impairment and dyslexia. As outlined above, many (30–50%) children with LLI also show impairments in auditory processing (e.g. Ramus 2003), despite normal sensitivity. A variety of explanations have been posited for the poorer performance of many children (and adults) with LLI on tests of auditory processing. These range from cognitive (e.g. attention, memory/processing capacity, e.g. Hanson & Montgomery 2002; Breier *et al*. 2003; Roach *et al*. 2004; Ahissar *et al*. 2006) to sensory (e.g. Tallal 2004) explanations. In an attempt to distinguish between these explanations, many studies have used electrophysiology (EEG/ERP). They have shown that, even during a ‘passive’ (unattended) hearing task, many children with LLI have atypical cortical (e.g. McArthur & Bishop 2005; Bishop 2007) and brainstem (e.g. Wible *et al*. 2005) evoked responses to speech and/or non-speech stimuli. These atypical neural responses, in the absence of cognitive engagement with the auditory stimulus, have been interpreted as supporting a sensory explanation of the listening problems associated with LLI. While it is tempting to conclude that abnormal auditory system physiology, particularly at such a low level as the brainstem, is indicative of ‘bottom-up’ processing, it is becoming increasingly clear that ‘top-down’ neural pathways can and do exert a major influence on all levels of the auditory system, even during general anaesthesia (e.g. Palmer *et al*. 2007). Descending systems, including those with origins beyond the classic central auditory system (e.g. frontal cortex), could have longer term modulating effects on brainstem or even on cochlear activity, as recently suggested in an adult human study showing that efferent olivocochlear activity predicts improvement in an auditory discrimination learning task (de Boer & Thornton 2008). How aberrant behavioural and physiological responses in children with LLI compare with those who have been diagnosed with APD remains to be studied.

### (d) Intervention models

Listening problems in children are traditionally managed in the same way as children referred with hearing problems. If there is no hearing loss, they may be sent away without any specific advice or treatment. Alternatively, they may be advised to improve their listening strategy or environment or they may be given an amplification device. It is likely that these latter forms of management are effective, but we are unaware of evidence that they have been specifically tested for children with listening problems (APD).

Auditory learning techniques have been applied in several studies to children with LLI (reviewed in §§1 and 2) and to adults with SNHL (e.g. Sweetow & Sabes 2007), but not yet to children with separately diagnosed APD. Owing to the likely close relation between the listening difficulties in APD and, when they occur, in LLI, many clinicians managing APD are currently recommending the use of auditory training. Scientific verification of this approach requires both a validated and agreed diagnostic framework for APD and further evidence for the efficacy of learning in providing benefit.

## 4. Auditory learning in adults

The clinical application of training in children has produced mixed results (see §§1 and 2). Reasons cited for this range from different implementation and deployment of training to differences in the chosen child populations in different studies. However, there is currently little evidence concerning the efficacy of various forms of training and, hence, the most optimal form of training. The overall improvement and the time course over which performance gain is observed depends on task-specific factors, such as task demands and task difficulty, as well as more general factors such as the regimen and content of the training sessions. Moreover, learning is influenced by motivation and cognitive factors such as attention (either task-specific or general arousal) and memory; factors that characterize ‘listening’ rather than just ‘hearing’ (see §3). Finally, while perceptual learning has traditionally been considered highly stimulus specific, the studies reviewed in §§1 and 2 suggest much more wide-ranging transfer of learning. Some of these factors have been addressed in recent adult studies of auditory learning, and will be described in this section. In §5, we discuss learning in children. Studies of auditory learning in adults can be divided into two categories. Most use auditory learning as a means of investigating the auditory system itself (e.g. Wright & Fitzgerald 2001; Delhommeau *et al*. 2002; Demany & Semal 2002; Fitzgerald & Wright 2005; Mossbridge *et al*. 2006). However, as outlined above, some are more concerned with searching for rules of learning and the variables that affect them. It is this category that we focus on here.

**Figure 3:**
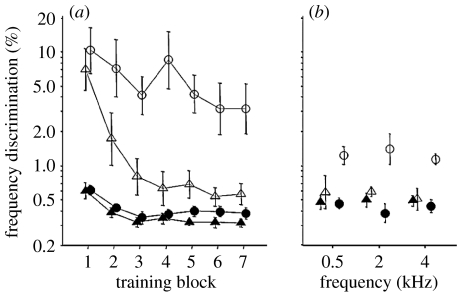
(*a*) Frequency discrimination learning curves for listeners with ‘better’ and ‘poorer’ initial performance, trained using a single (‘fixed’) standard frequency of 1 kHz or 5 different (‘varying’) standard frequencies (570, 840, 1170, 1600 and 2150 Hz) varied on a trial-by-trial basis. Frequency discrimination thresholds are presented as per cent difference between the standard and comparison (target) tone frequencies relative to the frequency of the standard. For listeners trained on varying frequencies, the results are averaged across frequencies (thresholds did not differ significantly between frequencies). (*b*) Transfer of learning tested at various untrained frequencies (each tested using a fixed standard frequency). Fixed: filled triangle, better; open triangle, poorer. Varying: filled circle, better; open circle, poorer. Figure adapted with permission from Amitay *et al*. (2005).

Learning of a simple perceptual task, such as pure tone frequency discrimination, can be fast (within a half hour of training) and dramatic (orders of magnitude change in performance). Auditory learning can be observed both within a single training session (Hawkey *et al*. 2004) and across multiple sessions (Amitay *et al*. 2005). As a rule, early learning is the most dramatic, with performance improvements becoming smaller over time. While it is often considered that early (‘single session’) learning is ‘procedural’, in that it reflects a familiarization primarily with the response demands of the task, we have demonstrated (Hawkey *et al*. 2004) that even very early learning, as seen in the frequency discrimination task shown in figure 3, can have a predominant ‘perceptual’ component.

The time course of learning is also strongly influenced by individual differences. For example, Amitay *et al*. (2005) found that when training on frequency discrimination where the standard tone was variable (ranging from 570 to 2150 Hz), and changed on a trial-by-trial basis, some listeners did not show the rapid learning observed with an unchanging standard, but rather showed slower improvement over a longer time (figure 3*a*). These listeners were differentiated from others by poorer initial performance on the task. They also showed reduced transfer to untrained frequencies (figure 3*b*). It is possible these ‘poorer listeners’ were using a less than optimal listening strategy that prevented efficient learning when the perceptual context required rapid shifts in attention between frequency bands.

**Figure 4:**
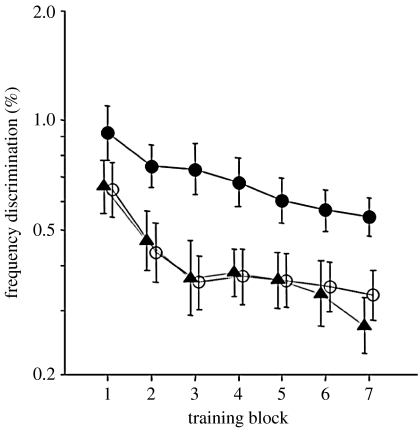
Frequency discrimination learning curves for listeners trained using a single, fixed standard frequency (filled triangles) of 1 kHz or 5 different frequencies (900, 950, 1000, 1050 and 1100 Hz) varied either on a trial-by-trial basis (filled circles) or a block-by-block basis (open circles). Frequency discrimination thresholds are presented as per cent of the standard (comparison) tone frequency. For listeners trained on varying frequencies, the results are averaged across frequencies (thresholds did not differ significantly between frequencies). Figure adapted with permission from Moore & Amitay (2007).

Even in better listeners, the way in which stimuli are presented within a training session can affect learning. Varying the standard stimulus by a small amount on a trial-by-trial basis lead to slow and protracted learning compared with training with an unchanging standard (Amitay *et al*. 2005). However, training on the same stimuli when they were blocked (each block used a different standard) did not differ from training with a single standard (figure 4; Moore & Amitay 2007). Thus, the method of presentation can affect training and transfer. Moreover, training on more than one task within a session can cause interference between tasks and impair learning (Wright *et al*. 2008).

While variations in the training set or task influence learning, it appears that learning is insensitive to the exact psychophysical procedure used. In comparing two- or three-interval trials, and two- or three-alternative choices within a trial, no differences were found in the pattern of early learning of a frequency discrimination task (Amitay *et al*. 2006*a*). This is perhaps surprising because, for a constant number of trials, a greater number of intervals would mean more exposure to the standard stimulus, so we might predict more learning would occur. Moreover, we might predict that an easy-to-difficult procedure, such as an adaptive staircase (Levitt 1971), would be preferable to a more volatile procedure, such as a maximum-likelihood estimator (Green 1995), owing to the gradual nature of increasing the difficulty and providing sufficient trials where the target can be easily detected. However, it turns out that the procedure has very little effect on early learning, even when using a constant set of stimuli that does not change adaptively—so long as the task remains challenging enough (see below; Amitay *et al*. 2006*b*).

**Figure 5:**
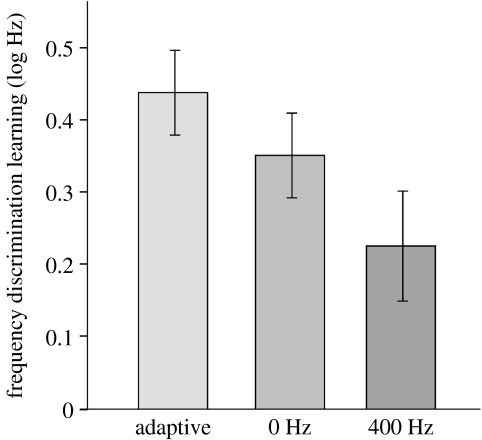
Frequency discrimination learning of a 1-kHz tone for 800 trials of frequency discrimination training using either adaptive tracking of the threshold at 75% correct (‘adaptive’), no difference between stimuli (0 Hz), or a large, fixed difference (400 Hz). Adapted with permission from Amitay *et al*. (2006*b*).

Based on the observations in visual learning (e.g. Ahissar 1999), it is generally considered that perceptual learning will not occur if the training task is too difficult (e.g. Cansino & Williamson 1997). Ahissar & Hochstein (2000) suggested that, when the task is too difficult, task-relevant information is inaccessible to the neuronal circuits attempting to perform it. We might also predict that learning will be suboptimal if the training task is too easy (see below). Learning would thus be optimal when training is kept at a difficult but possible level, sometimes referred to as the ‘edge of competence’. It has been suggested, alternately, that ‘easy-to-hard’ training would produce optimal learning (Liu *et al*. 2008), where the task-relevant information is made available to more and more specific neuronal populations as training progresses. The prediction regarding training on an ‘easy’ task has been borne out (Amitay *et al*. 2006*b*). When the standard and comparison sounds were kept so different during training that performance was at or near ceiling (100% correct), learning, though still significant, was reduced (figure 5), presumably because less engagement with the stimuli was required to perform the task successfully. However, the same study showed that very difficult tasks, and even an impossible task (attempting to discriminate identical sounds) resulted in robust learning of the discrimination. Signal detection theory suggests that, even when the stimuli are physically identical, they may appear perceptually discriminable due to the effect of internal noise (C. Micheyl 2008, personal communication). In any case, this result suggests that, while a training task can be made too easy to be effective, it apparently cannot be made too difficult. These findings also suggest that attention plays a fundamental role in learning: when the training task is challenging and requires a commitment of attentional resources it results in robust learning (Amitay *et al*. 2006*b*).

Transfer of learning to an untrained task is perhaps the most important issue from an applied perspective. It is often found that perceptual learning does not transfer between tasks, even when the training stimuli are very similar, or identical, for both tasks. For example, training in tone intensity discrimination was found not to transfer to frequency discrimination (Hawkey *et al*. 2004; Amitay *et al*. 2008), suggesting learning depends more on attending to a specific stimulus dimension than on adaptation or sensitization to the training stimulus. Similarly, in a study of auditory lateralization (Wright & Fitzgerald 2001), learning did not transfer between training using either interaural time or level difference cues, even though task instructions were identical and listeners were unaware of which cue was being trained or tested. However, in contrast to this apparently high specificity of auditory training, improvement in an auditory task (frequency discrimination) has been found following training on non-auditory tasks, such as Tetris (Amitay *et al*. 2006*b*; see below), and training on auditory tasks has been found to improve broader cognitive skills, such as memory (Mahncke *et al*. 2006). We can offer no simple or definitive explanation for these results. In the reverse hierarchy theory of visual learning (Ahissar & Hochstein 2004), it has been argued that behavioural improvement deriving from lower, more sharply tuned levels of the brain does not transfer to new stimulus conditions as readily as learning resulting from higher level brain plasticity. Similarly, training on easier visual tasks is thought to produce greater transfer than training on more difficult tasks. In the results cited above, these two forms of training may occur during different phases of adaptive learning. Typically in learning research, a single learning index encompasses both early and later phases of training. In addition, it is highly unusual in laboratory studies of perceptual learning for transfer of learning to different skills or modalities to be assessed. It is thus possible, in this variant of the reverse hierarchy theory, that the early easy stages of training contribute to the very broad, but lasting transfer observed, whereas later stages contribute to the specificity observed more commonly.

In §§1 and 2, we highlighted the importance of appropriate control groups for the interpretation of transfer of learning from training on simple auditory stimuli to language related skills. The transfer of learning from non-auditory tasks to auditory tasks discussed above is similarly dependent on the interpretation of control group results. Amitay *et al*. (2006*b*) examined this issue by comparing frequency discrimination learning induced by conventional, adaptive training, with the change in frequency discrimination over the same time frame in three control groups. Two of these groups played Tetris and showed significant frequency discrimination learning, relative to a ‘no-change’ baseline (single sample *t*-test). However, neither of the Tetris training groups (one of whom also listened passively to tones while training) improved significantly in frequency discrimination relative to the third, ‘waiting-room’ control group (see §§1 and 2). While the latter group did not show significant learning, relative to baseline, their mean performance on the frequency discrimination task did improve slightly.

We have now conducted several studies in which we have found that waiting-room control groups, at least in a simple frequency discrimination task, gain a small amount of learning from performing the probe test of frequency discrimination, as suggested in §§1 and 2. Based on our repeated findings of learning resulting from small numbers of trials, or otherwise minimal task exposure, it can thus be more appropriate to compare the performance of a trained group with a no-change baseline than with a control group who are tested with, trained on, or exposed to, another task. It depends on the hypothesis which, in turn, depends on the purpose of the study. For studies, such as that of Amitay *et al*. (2006*b*), whose purpose is to dissect the contributions to training, we suggest that the no-change baseline is the correct one. In most studies of auditory learning (e.g. Wright *et al*. 1997; Delhommeau *et al*. 2002), much longer periods of testing have been used before training commences. Our data suggest that such a design risks confusion between learning obtained during the initial period of testing with that obtained during the designated training period. The issue of transfer of auditory learning is of such immediate translational importance that it is clearly one in need of further research.

Another important issue that, surprisingly, is yet to be systematically addressed in controlled experiments, is retention of auditory learning over varying periods of time. Tallal *et al*. (1996) reported retention of speech and language gains for at least six weeks following the end of mixed auditory training (details in §§1 and 2), and we (Moore *et al*. 2005; figure 1) found retained improvement on language measures for at least five weeks following phoneme discrimination training. It should be noted that neither of these studies controlled for repeated language testing in an untrained group. However, retention of the trained task has been shown for comparable times. Thus, significant frequency discrimination learning was retained for at least two months after multiple session training (S. Amitay, D. J. C. Hawkey & D. R. Moore 2005, unpublished data). Wright and her colleagues have reported several instances of learning retained for one month after multiple session training and, anecdotally, two participants for whom amplitude modulation (AM) rate discrimination learning was retained, and AM detection learning was lost, a full 15 months after one week of training (Fitzgerald & Wright 2005). It therefore appears that retention over at least one to two months is the norm, with much longer term retention a possibility worthy of further investigation. But many questions remain unanswered. It is, for example, unclear whether a shorter or single training session is sufficient for long-term retention. It has been shown that a minimum amount of temporal interval training within a session is necessary for learning to be retained until the next day (Wright & Sabin 2007), but not whether additional trials or sessions are necessary for the learning to be retained long term.

When considering these ‘rules’ of auditory learning, it needs to be kept in mind that the reviewed evidence has relied on comparing average performance for groups of listeners. Individuals whose performance lies outside the ‘norm’ are often excluded from investigation. Individual variability in naive (untrained) auditory performance plays a significant role in the learning pattern (figure 3*a*), as well as in the pattern of transfer between tasks (figure 3*b*). This variability is of particular importance as we go on to consider children of different ages, as well as different abilities, in §5. Finally, the summary statements presented above have been derived from data on a limited range of stimuli (mainly tones) and tasks (mainly frequency discrimination). It seems almost certain that many of these ‘rules’ will be strongly influenced, at least quantitatively, by the selection of training and testing materials.

## 5. Auditory learning in children

Despite a growing body of information regarding the mechanisms and rules underlying auditory learning in adults, very few studies have investigated these issues in children. This is surprising, given the emergence (see §§1 and 2) of auditory training programmes (e.g. Fast ForWord, Earobics (http://www.earobics.com/solutions/programs.php), Phonomena) that are aimed at improving listening skills, and which are targeted primarily at the child market. Given that observations derived from adults may not apply to children, it seems timely that we make efforts to understand the processes underlying auditory learning during development, and the extent to which these transfer to other cognitive abilities. To our knowledge, we have conducted the first and only laboratory-based experimental test of (non-speech) auditory learning in typically developing children (Halliday *et al*. 2008). In this section, we review the findings of this study to outline some of the issues that are important when assessing auditory learning in children, and to illustrate some of what we do (and do not) know about this topic.

**Figure 6:**
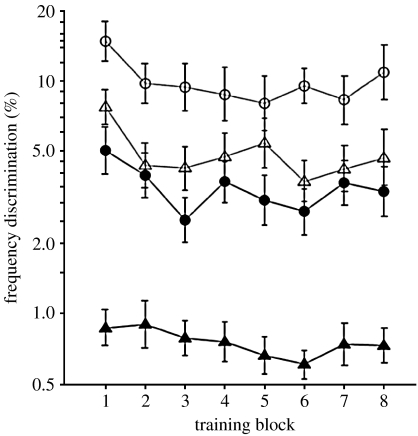
Frequency discrimination learning curves for 6- to 11-year-old children and adults trained using a single fixed standard frequency of 1 kHz. Frequency discrimination thresholds are presented as per cent of the standard (comparison) tone frequency. Open circle, 6–7 years; open triangle, 8–9 years; filled circle, 10–11 years; filled triangle, 18+ years. Adapted with permission from Halliday *et al*. (2008).

Halliday *et al*. (2008) examined the effects of age on auditory learning, by giving 6- to 11-year-old children and adults approximately 1 hour of training on a frequency discrimination task. We found (figure 6) that children on average had poorer frequency discrimination skills compared with adults at the outset of training, although performance improved with age. We showed that it was possible to induce auditory learning in children, even in those as young as 6 years of age. Nevertheless, across age groups, learning was confined to early training blocks (approx. 200 trials). We do not know whether the training in later blocks that occurred without measurable learning was in any way beneficial. This is an important question, as the optimization of auditory training packages is likely to be particularly crucial for children, where compliance to regimes that are, by definition, repetitive, may be an issue.

**Figure 7:**
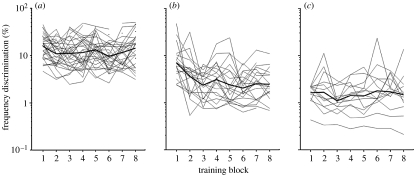
Individual and group frequency discrimination learning curves for (*a*) ‘Non-adult-like’ children who did not achieve adult-like frequency discrimination thresholds at any point during the training session, (*b*) ‘Trainable’ children who did not have frequency discrimination thresholds at the outset of training, which were similar to those of naive adults but nonetheless went on to achieve this during the training session (thin line, individual; thick line, group mean) and (*c*) ‘Adult-like’ children who had frequency discrimination thresholds at the start of training, which were similar to those of naive adult listeners. Adapted with permission from Halliday *et al*. (2008).

Our data also highlight the considerable individual differences in performance at the outset of training, particularly in child groups. While these differences partly reflect a common feature of our approach to provide only minimal exposure to a task prior to data collection, they are also of theoretical interest. Figure 7 shows the child data divided according to frequency discrimination thresholds over the course of training. Twenty per cent of the children had thresholds at the start of training that were comparable to those of naive adult listeners, and were denoted the ‘adult-like’ subgroup. A further 30 per cent of children went on to achieve adult-like thresholds during the training session. These were denoted the ‘trainable’ subgroup. The remaining 50 per cent of children did not achieve adult-like thresholds and were denoted the ‘non-adult-like’ subgroup. Subgroup membership was linked to the interplay between three different factors: age; non-verbal IQ; and attention. Adult-like children tended to be older, had slightly above average non-verbal IQ and showed fewer ‘attention lapses’ (the extent to which performance fell short of 100 per cent correct at the highest Δ*F*). The trainable subgroup was similar in age and IQ to the adult-like group, but showed a greater number of attention lapses. Finally, the non-adult-like group was younger, had poorer attention than both the other groups, and had lower IQ than the adult-like group. These findings suggest that both initial (naive) performance, and learning, may be dependent upon the child's level of cognitive maturation.

The results of this study (Halliday *et al*. 2008) illustrate two additional general points. First, when children and adults produce comparable performance, the underlying processes may not be the same. Unlike adults, the adult-like child listeners did not, as a group, show subsequent learning with training, despite performing relatively well at the start of training. Second, our child listeners did not show transfer of learning to an identical task with a different standard frequency. Task learning and transfer of learning in adults can follow different time courses (Wright & Sabin 2007), and it is therefore possible that we might see greater transfer in children if they are trained over a longer time. Another possibility is that greater stimulus variability is required for successful transfer of learning. Clearly, if auditory training programmes are to be of applied relevance, finding the answers to these and other questions should be a priority.

## 6. Summary and conclusions

Auditory learning is as natural as breathing; most of us do it all the time. But when the natural process is disrupted by a peripheral or central disorder of the auditory system, an intervention beyond the restoration of sufficiently amplified input may be helpful. At the point at which conventional remediation ceases to be effective, or as a supplement or alternative to that remediation, training may prove to be a useful way to achieve a more complete restoration of function. Training may also offer a less labour intensive and hence more cost-effective form of intervention. The value of using laboratory-based directed training to promote performance on almost any auditory task is well recognized and we have presented here some advances in understanding the basic rules and limitations of this type of training. The challenge now is to harness the potential for training to become a useful tool in the management of a variety of auditory-based disorders.

We have demonstrated that learning an auditory task can be very fast and very dramatic. These factors depend on the properties and variability of the training stimuli, and the manner in which they are presented. They are subject to individual differences in auditory processing ability and a host of other factors. Auditory learning is the greatest when an auditory training task is relevant, challenging and engaging, but learning may be induced to some degree by simply performing an engaging, though apparently unrelated task. Prediction of transfer between tasks is non-trivial and can, for example, be asymmetric, even when using identical stimuli for training. Understanding transfer is crucial for the application of training in clinical and other applied contexts. The detailed interpretation of experimental control conditions is, in turn, crucial to this understanding.

Initial results with children suggest that training can produce measurable improvements in both a trained task and in more general listening and language skills. However, few well-controlled studies have been performed, and some have provided less encouraging results. One difficulty is that complex training regimens have led to results that are difficult to interpret theoretically and to optimize practically. Optimizing training is certainly a priority when working with children, as it is so difficult to engage their attention over long or multiple training sessions. The potential for alleviating a variety of language and auditory-based disorders makes it imperative that we do so.

There is considerable evidence that auditory training can be an effective intervention for a variety of auditory-based disorders and problems, not only from those arising in early childhood, but also for the decline of hearing in middle- and old age. The type of training used and the duration and frequency of training sessions should, of course, depend on the target group, in terms of age, disabilities (both auditory and non-auditory), and purpose for which the training is designed. However, sensory training is not a panacea, and a realistic outlook on what we might expect training to achieve in the context of an overall educational or patient management context is key to its successful application.
